# How active social network site use affects green consumption: A moderated mediation model

**DOI:** 10.3389/fpsyg.2023.1124025

**Published:** 2023-03-15

**Authors:** Yanping Gong, Chunyan Chen, Yuxuan Tan, Danni Tang

**Affiliations:** ^1^School of Business, Central South University, Changsha, China; ^2^School of Business Administration, Hunan University of Finance and Economics, Changsha, China

**Keywords:** active SNS use, public self-awareness, green consumption, impression management motivation, self-awareness theory

## Abstract

A growing body of literature suggests a link between the usage of social networking sites (SNSs) and green consumption. However, researchers have shown that not all types of SNS usage have the same effect on individuals; therefore, to fully understand the relationship between a particular SNS use type and green consumption, as well as the mechanisms underlying the relationship, more research is required. This study examined a moderated mediation model based on self-awareness theory to explain the “how” and “why” of the relationship between active SNS use and green consumption. An offline survey (*N* = 210) and an online survey (*N* = 348) were conducted. The results suggest that active SNS use is positively associated with green consumption *via* public self-awareness and that impression management motives moderate the mediating role of public self-awareness in the relationship between active SNS use and green consumption. By examining the connection between a specific type of SNS use (active SNS use) and green consumption, our study adds to the body of literature on the causes of green consumption. The results have substantial implications for future research promoting socially responsible consumption behavior.

## Introduction

The consumption of environmentally friendly goods and services has become popular over the last decades and has been growing continuously (Ge et al., 2020; Kumar et al., 2021; Zhao et al., 2021). Green consumption, as a representative pro-environment behavior (Lee et al., 2014), is defined as the tendency to express environmental protection values through purchasing and consuming behaviors (Haws et al., 2014). Several studies demonstrated the benefits of green consumption for individuals, societies, and the environment (Li et al., 2022). Specifically, customers with green buy intent and behavior scored more highly on the life satisfaction scale (Xiao and Li, 2011; Dhandra, 2019). The degree of customer desire for green products is always positively correlated with overall societal welfare (Zhang et al., 2019). Moreover, green consumption helps solve the problems of the over-exploitation of natural resources (Nguyen et al., 2019) and controlling air pollution (Zhang et al., 2019). Therefore, it is essential to investigate the causes and the contributing factors of green consumption to induce consumers to go green (Fachrurazi et al., 2022).

In recent years, productive research has been conducted on the antecedents of green consumption, primarily personal attributes and external contexts. Personal attributes factors include self-construal (Mancha and Yoder, 2015), regulatory focus (Miniero et al., 2014), social responsibility consciousness (He et al., 2016), and moral identity (Wu and Yang, 2018). In contrast, external contexts include message framing (Amatulli et al., 2019), social exclusion (Guo et al., 2020), and packaging color (Felix et al., 2021). Beyond these factors, SNS use has also recently begun to attract researchers’ attention. Social media has become an important way for people to interact (Verduyn et al., 2020; Wang et al., 2020; Lau et al., 2022). As of 2021, there were more than 4.2 billion active social media users worldwide. By 2027, that number is projected to rise to 6 billion (Dixon, 2022). In turn, SNS use has had significant psychological and behavioral repercussions on individuals (e.g., Han et al., 2020; Pepper et al., 2022; Reed, 2023). Hence, more and more researchers have begun to explore the impact of SNS use on green consumption. For example, SNS use and online interpersonal influence were shown to be positively related to green purchase intentions among millennials in the United States (Bedard and Tolmie, 2018). SNS use was found to be associated with positive attitudes toward green cosmetics (Pop et al., 2020). More recently, SNS use was found could impact sustainable purchasing attitudes *via* the drive for environmental responsibility (Zafar et al., 2021).

However, previous studies have only explored the impact of general SNS use on green consumption. They have yet to consider the disparities across the various types of SNS use. Recent research on SNS use and its impact suggested that not all social networking activities are equally social and that there are significant differences in the effect of different SNS use types on people’s attitudes and behaviors (Frison and Eggermont, 2016; Thorisdottir et al., 2019; Ng, 2020; Yue et al., 2022). These studies implied that when analyzing the effects of SNS use, we must focus on specific types of SNS use to draw valid conclusions (Frison and Eggermont, 2016; Verduyn et al., 2021).

Existing research classifies SNS use into two types: active SNS use and passive SNS use (Pagani et al., 2011). Active SNS use refers to the activities of exchanging information directly with other SNS users (Verduyn et al., 2017, 2021). Passive SNS use refers to simply consuming information posted by other people without directly communicating with them (Verduyn et al., 2017, 2021). Active SNS use could have positive consequences such as more social closeness (Neubaum and Krämer, 2015), higher social support (Frison and Eggermont, 2016), higher self-esteem (Lin et al., 2020), and higher subjective well-being (Verduyn et al., 2021). In contrast, passive SNS use leads to increased envy (Ding et al., 2017), increased loneliness (Burke et al., 2010), and more severe depression (Frison and Eggermont, 2016), which are antithetical to positive connections with others. Since green consumption is a positive altruistic behavior advocated by the public (Lu et al., 2013; Zhang et al., 2019), it is reasonable to construct the relationship between active SNS use (neither passive SNS use nor general SNS use) and green consumption. Unfortunately, as far as we know, there has yet to be an empirical study on the connections between active SNS use and green consumption. To address this research gap, we investigated the effect of active SNS use on green consumption and the explanatory mechanisms and boundary conditions involved.

Self-awareness theory (Duval and Wicklund, 1972) helps explain active SNS use and green consumption. Self-awareness theory suggests that the presence of an audience causes a person to calculate the difference between his or her current image and the expected image and to try to present the expected self (Leary and Kowalski, 1990; Carver and Scheier, 2001). When people frequently interact on SNS, their awareness of the audience’s presence and public self-awareness could be enhanced (Froming et al., 1982; Tu and McIsaac, 2002). In response to this awareness, people may behave in ways that are more consistent with societal expectations (Duval and Wicklund, 1972; Carver and Scheier, 2001). As a pro-environment behavior, green consumption has been a consumer trend advocated by the public in the last decade (Lu et al., 2013; Zhang et al., 2019). As active SNS users become self-aware, they may be more inclined to engage in green consumption.

**Figure 1:**
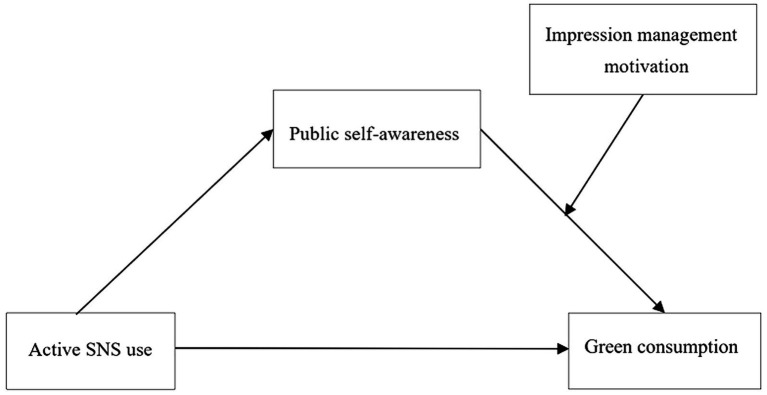
Overview of the proposed moderated mediation model.

Therefore, the purposes of this study were to examine (a) whether active SNS use is linked to green consumption, (b) whether public self-awareness mediates the relationship between active SNS use and green consumption, and (c) whether impression management motivation moderates the relationship between active SNS use and green consumption. Thus, we established and tested a moderated mediation model (Figure 1). We used regression-based analyzes in two independent studies to test the hypothesized model. Our research contributes to the literature on SNS use and green consumption by examining the impact of specific type of SNS use (active SNS use). Meanwhile, this research has significant implications for governments’ promotion on pro-environment behavior, companies’ green consumption marketing efforts and for consumers to understand their consumption behavior.

## Theoretical Framework and hypotheses

### Self-awareness theory

We can draw on self-awareness theory (Duval and Wicklund, 1972) to build the conceptual framework of this research. When a person’s attention is directed inward and triggered by the situation, it is called objective self-awareness (Yue et al., 2022). There are two kinds of self-awareness: public self-awareness and private self-awareness (Froming et al., 1982). These two types of self-awareness are susceptible to be impacted in distinct settings. For example, being recorded on video or having an audience can enhance public self-awareness, and looking in a mirror or keeping a diary can increase private self-awareness (Scheier and Carver, 1980; Froming et al., 1982). Behaviors that reflect personal attitudes may stem from a focus on the private self, while behaviors that reflect social expectations may stem from a focus on the public self (Froming et al., 1982). Researchers have used audiences to activate public self-awareness and make the individual’s social attributes more prominent, thereby inducing regulatory behavior to match the audience’s expectations (Scheier and Carver, 1980; Froming et al., 1982). When aware of an audience, the person compares themselves with the audience and if there is a discrepancy, they may actively change their behavior to be more congruent with the social standards (Duval and Wicklund, 1972; Carver and Scheier, 2001).

Previous studies confirmed the applicability of self-awareness theory in the context of social networking. Drawing on the theory of self-awareness, Marder et al. (2016) posited that the impact of SNS surveillance might make people feel monitored by audiences even in real life. Furthermore, Lavertu et al. (2020) found that the offline salience of online audiences will increase people’s public self-awareness and external motivation, thus increasing people’s willingness to participate in offline prosocial activities.

### Active SNS use and green consumption

Active SNS use involves sending messages, updating one’s status, and posting photos in private or public channels (Frison and Eggermont, 2016). Active SNS use increases interpersonal interactions and communication and contributes to creating and developing relationships (Liu et al., 2020). Therefore, active SNS use will allow people to build more peer relationships than just passively browsing for information (Neubaum and Krämer, 2015; Thorisdottir et al., 2019). Compared to passive SNS use, which increases loneliness (Burke et al., 2010), active SNS use increases social support and attention from others (Frison and Eggermont, 2016). In addition, a previous study has pointed out that people who actively interact on SNS have the motivation to increase social capital (Burke et al., 2011), so they have a strong sense of audience when actively interacting on SNS. This is why consumers who actively share a zero-waste lifestyle (ZW lifestyle) on social media are able to adhere more to this lifestyle (SÄƒplÄƒcan and Márton, 2019).

Therefore, people will be aware of audience presence when interacting actively on SNS, and their sense of public self will increase accordingly (Tu and McIsaac, 2002). According to self-awareness theory, focusing on the public self may lead to behavior more aligned with social norms (Duval and Wicklund, 1972). Consequently, people who actively use SNS are more willing to conform to subjective norms than passive SNS users. Green consumption is an environmentally friendly behavior that the government and the public have advocated in recent years (Lu et al., 2013; Zhang et al., 2019), and it can help consumers develop caring and ethical social images (Griskevicius et al., 2010). Therefore, we hypothesize that people who are active SNS users will be more willing to engage in green consumption.

*H1*: Active SNS use will be positively associated with green consumption.

### The mediating role of public self-awareness

Public self-awareness may mediate the relationship between active SNS usage and green consumption. Public self-awareness is the degree to which individuals worry about what others think of them (Duval and Wicklund, 1972; Govern and Marsch, 2001). Therefore, when the perceived audience increases, people’s public self-awareness will increase accordingly (Froming et al., 1982; Park et al., 2022). Conventional psychological researchers have used actual or perceived audiences to activate participants’ public self-awareness (Scheier and Carver, 1980; Froming et al., 1982). Nowadays, SNS is a common way for people to communicate with a wide range of audiences (Cocea, 2017). Specifically, active SNS use, such as chatting with others or updating their status online, could enhance the perception of audience presence (Tu and McIsaac, 2002; Deters and Mehl, 2013; Ranzini and Hoek, 2017). Thus, active SNS use may be positively associated with public self-awareness.

The relationship between public self-awareness and green consumption has been well established. For instance, public self-awareness was shown to contribute to consumers’ green restaurant consumption (Hwang and Lee, 2019). When consumers are publicly accountable, they are more responsive to other-benefit appeals and have higher purchase intentions for environmentally friendly consumption (Green and Peloza, 2014). According to self-awareness theory (Duval and Wicklund, 1972), individuals with high public self-awareness adjust their behaviors to meet the public’s expectations. Green consumption is an altruistic behavior that conforms to social norms (Lu et al., 2013; Zhang et al., 2019), and active SNS users may engage in this behavior to reduce a discrepancy between self-awareness and public expectations.

In sum, active SNS use increases awareness of the audience’s presence (Tu and McIsaac, 2002; Marder et al., 2016; Lavertu et al., 2020), creating a higher public self-awareness. In turn, higher public self-awareness promotes behavior by public expectations (Froming et al., 1982), leading to more green consumption.

*H2*: The relationship between active SNS use and green consumption will be mediated by public self-awareness.

### The moderating role of impression management motivation

Active SNS use affects not everyone equally or to the same degree (Zheng et al., 2022). The relationship between public self-awareness and green consumption may vary from person to person. Not all people will make green purchases after active SNS use has stimulated public self-awareness, depending on whether they are motivated by impression management (Leary and Kowalski, 1990). Impression management involves impression motivation and impression construction (Leary and Kowalski, 1990). Impression management motivation is the desire to manage how one comes across to others (Goffman, 1959; Leary and Kowalski, 1990). Once people are motivated to create a certain impression, they are likely to perform certain actions to construct that image (Leary and Kowalski, 1990). For example, they may act in ways that are socially desirable to make a good impression (White and Peloza, 2009). Conversely, people with low impression management motivations will be less concerned about what others think of them. Accordingly, they are less likely to make efforts to improve the way others perceive them.

Research on impression management points out that people tend to make positive impressions of others, including the image that they possess solid prosocial attributes (Leary, 1986; Zhang et al., 2019). Meanwhile, green consumption can help consumers develop a social image of someone caring and ethical (Griskevicius et al., 2010). Like impression management theory, self-awareness theory suggests that when people are aware of an audience’s presence, they may adjust their behavior to conform to social norms and expectations (Duval and Wicklund, 1972; Carver and Scheier, 2001). Thus, with increased heightened self-awareness, individuals with high impression management motivation may be more likely to engage in green consumption than those with low impression management motivation.

Thus, it can be assumed that impression management motivation can positively moderate the effect of public self-awareness on green consumption. Specifically, for people with high (vs. low) motivation for impression management, the self-awareness that comes from active SNS use has a stronger impact on green consumption.

*H3*: The mediation effect of public self-awareness in the relationship between active SNS use and green consumption will be moderated by impression management motivation. Specifically, this mediation effect will be stronger for individuals with high (vs. low) impression management motivation.

## Overview of studies

Two studies were conducted to investigate the hypothesized links. In Study 1, we conducted a preliminary survey study by paper-and-pencil questionnaires to test the proposed relationship between active SNS use and green consumption and the mediating role of public self-awareness. Study 2 was devised to replicate these results using online questionnaires and test the moderator of impression management motivation in the mediation role of public self-awareness. The two studies used different measures of active SNS use (two-dimensional or unidimensional) and green consumption (intentions or behaviors).

Active SNS use has been considered a two-dimensional construct in some research and a one-dimensional construct in others. As a two-dimensional construct, active SNS use includes both active public and private SNS use (Frison and Eggermont, 2016; Liu et al., 2020). Active public SNS use refers to the users’ interactions with SNS friends in a public context (e.g., status updates and posting photos). Active private SNS use refers to private interactions between the user and SNS friends (e.g., sending private messages; instant messaging). However, researchers who support the idea of a one-dimensional construct question the public/private distinction in any cumulative self-reported measure and point out that privacy settings can vary across users, platforms, and posts (Erliksson et al., 2020). Thus, in Study 1, we used a two-dimensional scale to measure active SNS use, while in Study 2, we used a unidimensional scale. It is believed that the combination of two active SNS use scales can provide sufficient support for the findings.

Moreover, in addition to active SNS use, Study 1 and Study 2 also used different scales to measure green consumption. Previous studies have used either the green consumption intentions or behavior scale to measure green consumption (Lan and Sheng, 2014; Nguyen et al., 2019; Sun et al., 2019). One problem evident in the research on green consumption is the gap between attitudes and behaviors, meaning that green consumption values or intentions often could not translate into green consumption behaviors (Nguyen et al., 2019). Although intentions and behaviors are closely related, they are not precisely equivalent (Ajzen et al., 2018). Therefore, we used green consumption intentions and green consumption behaviors as dependent variables in two studies, respectively. Study 2 was meant to replicate and expand Study 1’s findings from green consumption intentions to green consumption behaviors. Specifically, in study 1, we tested the relationship between active SNS use and green consumption intentions. In study 2, we tested the relationship between active SNS use and actual green consumption behaviors.

The scales used in these two studies have been shown to have good psychometric properties and have been widely used in previous studies. The English scales were translated into Chinese using the translation and back-translation method.

## Study 1

### Methods

#### Participants and procedure

The institution of the first author’s research ethics committee approved the current work. Data were collected from Chinese consumers in shopping malls. Participants gave informed consent and were assured that the survey was anonymous. After reading an explanatory statement that briefly summarized the aim of the study, participants completed paper-and-pencil questionnaires. After the research, each participant received a modest token of appreciation for their time and effort.

We recruited 263 consumers to participate in the investigation using convenience sampling. The total sample consisted of 210 consumers who submitted valid responses, with a response rate of 79.85%. Of the 210 consumers (54.29% female) in the final sample, the majority of participants were between 18 and 24 years old (59.52%), followed by the 25–30 age group (25.24%). The majority of participants was pursuing or had a junior college or bachelor’s degree (60%). Moreover, 68.57% of the participants earned below 50,000 RMB annually (about 7,169 U.S. Dollars), and 25.24% of the participants earned from 50,000 to 150,000 RMB annually (7,169 to 21,506 U.S. Dollars).

#### Measures

A measure of active SNS use developed by Frison and Eggermont (2016) was adapted for use in the current study. In the initial scale, the platform name “Facebook” was changed to WeChat, a social networking site that is more popular in China. Active public SNS use was assessed with three items such as, “How often do you post a message on your own WeChat Moments timeline?” Active private SNS use was assessed with two items, one of which was, “How often do you send someone a personal message on WeChat?” The ratings for each item ranged from 1 (never) to 7 (several times per day) on a 7-point Likert scale. The Cronbach’s α was 0.96 and 0.84 for active public and private SNS use, respectively. The Cronbach’s α of the overall active SNS use scale was 0.83.

Public self-awareness was measured by the revised Self-Awareness Scale (Govern and Marsch, 2001), including three items such as, “I am concerned about the way I present myself,” “I am self-conscious about the way I look,” and “I am concerned about what other people think of me.” Participants were instructed to score each statement on a 7-point Likert scale, with 1 for “strongly disagree” and 7 for “strongly agree.” In this study, Cronbach’s α was 0.85.

A scale developed by Lee et al. (2014) was used to measure green consumption intentions. This six-item scale included items such as, “I am considering purchasing products that are less environmentally harmful” and “I am planning to purchase products that are made by an eco-friendly business.” Participants were instructed to score each statement on a 7-point Likert scale, with 1 for “strongly disagree” and 7 for “strongly agree.” In this study, Cronbach’s α was 0.82.

Control variables include three variables. First, as women are more likely to be environmentally conscious and to scrutinize products advertised as sustainable compared with men (Kassinis et al., 2016), gender (0 = male and 1 = female) was controlled. Second, since education was found to be positively associated with green consumption (Sun et al., 2019), education level (1 = junior high school and below; 4 = master level and above) was controlled. Finally, personal annual income (1 = 50,000 RMB and below; 7 = 500,000 RMB and above) was also controlled because income was found to be positively associated with environmental perceived validity (Sun et al., 2019).

#### Analysis strategy

Study 1 used PROCESS macro (Hayes, 2013) in SPSS 24.0 to test hypotheses. SPSS PROCESS model 4 was used. 5,000 iterations of bootstraps generated the bootstrap-based 95% confidence intervals with bias correction for simple effects.

### Results

Table 1 displays descriptive statistics and correlations among variables. The correlations among active SNS use, public self-awareness, and green consumption intentions were significant. These results offer preliminary support for H1 and H2.

**Table 1:** Means, standard deviations and correlations among study variables in Study 1.

Variables	*M*	SD	1	2	3	4	5	6
1. Gender	0.47	0.55	-					
2. Education level	3.18	0.69	0.11	-				
3. Income level	1.60	1.07	0.08	0.10	-			
4. Active SNS use	4.75	1.00	−0.07	0.06	0.16^*^	-		
5. Public self-awareness	4.91	1.20	0.01	0.06	0.02	0.37^***^	-	
6. Green consumption intentions	5.08	0.81	0.02	0.16^*^	0.07	0.41^***^	0.33^***^	-

*N* = 210; ^*^p < 0.05, ^***^ *p* < 0.001.

Then, we did a confirmatory factor analysis to assess the discriminant validity of four self-reported measures. We compared two measurement models: the one-factor model and the hypothesized four-factor model. In the one-factor model, all of the items were loaded onto one factor. For the hypothesized four-factor model, items were loaded onto their respective hypothetical constructs. The results demonstrated that the fit index of the model was better when items were loaded onto their respective constructs (*χ*^2^/df = 1.49, CFI = 0.98, TLI = 0.97, IFI = 0.98, RMSEA = 0.05) than when all items were loaded onto one factor (*χ*^2^/df = 11.94, CFI = 0.50, TLI = 0.41, IFI = 0.51, RMSEA = 0.23). These results suggest that the measures in the model represented distinct constructs.

Table 2 (Equation 1) shows a positive relationship between active SNS use and green consumption intentions (*B* = 0.33, *p* < 0.001), H1 was supported. Both the total effect [effect = 0.33, 95% CI = (0.23, 0.43)] and the direct effect [effect = 0.27, 95% CI = (0.16, 0.37)] of active SNS use on green consumption intentions were positive and significant.

**Table 2:** Regression results of MODEL 4 in Study 1.

Variables	Equation 1 (Green consumption intentions)		Equation 2 (Public self-awareness)		Equation 3 (Green consumption intentions)	
	*B*	SE	*B*	SE	*B*	SE
Control variables						
Gender	0.05	0.09	0.09	0.14	0.03	0.09
Education level	0.15^*^	0.08	0.06	0.11	0.15^*^	0.07
Income level	−0.01	0.05	−0.05	0.07	−0.004	0.05
Independent variable						
Active SNS use	0.33^***^	0.05	0.45^***^	0.08	0.27^***^	0.06
Mediator						
Public self-awareness					0.14^**^	0.05
*R* ^2^	0.43^***^		0.38^***^		0.47^***^	

*N* = 210; ^*^p < 0.05, ^**^ *p* < 0.01, ^***^ *p* < 0.001.

As for H2, the mediation effect of public self-awareness in the association between active SNS use and green consumption intentions was statistically significant [effect = 0.06, 95% CI = (0.02, 0.12)]. Specifically, as shown in Table 2, there was a significant and positive relationship between active SNS use and public self-awareness (Equations 2, *B* = 0.45, *p* < 0.001), as well as between public self-awareness and green consumption intentions (Equation 3, *B* = 0.14, *p* < 0.01). Thus, the mediation effect of public self-awareness was identified, supporting H2. Taken together, the results provided support for H1 and H2.

## Study 2

### Methods

#### Participants and procedure

Data for Study 2 were collected online using the Sojump website, which is one of the most popular online survey websites in China, similar to SurveyMonkey in America. The authors recruited a subset of participants by posting advertisements on their social networking accounts (e.g., WeChat, QQ, and Microblog) and by commissioning a professional questionnaire collection agency in China. After completing the questionnaire, participants were told that they would receive a small payment for participating (approximately $0.30 U.S. Dollars), and they could send the link to the questionnaire to their friends if they wanted. The research ethics committee at the institution of the first author authorized the current work. Participants gave informed consent and were assured that the survey was anonymous. The final valid sample comprised 348 consumers, resulting in a valid response rate of 75.32%. Of the 348 consumers, 52.30% were female. Most participants were between 18 and 24 years old (60.06%) and had a junior college or bachelor’s degree (81.03%). Moreover, 42.53% of the participants earned below 50,000 RMB annually (about 7,169 U.S. Dollars), and 41.38% of the participants earned from 50,000 to 150,000 RMB annually (7,169 to 21,506 U.S. Dollars).

#### Measures

Whereas in Study 1 we measured active SNS use as two dimensions (public and private), in Study 2, we used a unidimensional measure named the Active SNS Use Questionnaire (ASUQ; Ding et al., 2017) to measure the frequency of active SNS use under various platforms (e.g., WeChat, QQ, Microblog) in the Chinese context (Lin et al., 2020). The measure consists of five items, such as: “I interact with friends when browsing their SNS (e.g., WeChat, QQ, Microblog) profile pages.” Respondents rated on a 5-point Likert scale according to the frequency of each behavior occurrence, with 1 for “never” and 5 for “very often.” In this study, the Cronbach’s α was 0.83.

Public self-awareness was measured using the same scales used in Study 1. The Cronbach’s α coefficient for public self-awareness in Study 2 was 0.72.

Study 2 used a different measure of green consumption than the measure used in Study 1. Specifically, the measure used in Study 2 assessed green consumption behaviors rather than green consumption intentions. The five-item scale (Kim and Choi, 2005) assessed the extent to which the participant purchased green products, as an indicator of actual green consumption behaviors. Example items are “I make a special effort to buy paper and plastic products that are made from recycled materials” and “I have avoided buying a product because it had potentially harmful environmental effects.” Participants were asked to rate their agreement on a 5-point scale, with 1 for “never” and 5 for “very often.” In this study, the Cronbach’s α was 0.82.

Impression management motivation was assessed by a nine-item scale (White and Peloza, 2009). Example items are “I want to make a positive impression on others” and “I want to make myself look good to others.” Participants were asked to rate their agreement on a 7-point Likert scale, with 1 for “strongly disagree” and 7 for “strongly agree.” The Cronbach’s α in the present study was 0.90.

The control variables were the same as those used in Study 1, namely gender, education level, and personal annual income.

#### Analysis strategy

Study 2 used PROCESS macro (Hayes, 2013) in SPSS 24.0 to test hypotheses. SPSS PROCESS model 4 and 14 were used. Five thousand iterations of bootstraps generated the bootstrap-based 95% confidence intervals with bias correction for simple effects. Aiming to replicate the findings of Study 1, study 2 used different measures of active SNS use and green consumption, and in addition tested the moderating role of impression management motivation.

### Results

Table 3 displays the descriptive statistics and correlations for each variable in Study 2. All of the study variables had substantial relationships. These results preliminary supported our hypotheses.

**Table 3:** Means, standard deviations and correlations among study variables in Study 2.

Variables	*M*	SD	1	2	3	4	5	6	7
1. Gender	0.48	0.50	-						
2. Education level	3.07	0.46	−0.11^*^	-					
3. Income level	2.17	1.27	0.22^***^	−0.03	-				
4. Active SNS use	3.49	0.70	0.02	−0.03	0.12^*^	-			
5. Public self-awareness	5.75	0.81	−0.02	−0.01	0.10	0.46^***^	-		
6. Impression management motivation	5.87	0.72	−0.04	−0.003	0.14^*^	0.42^***^	0.75^***^	-	
7. Green consumption behaviors	3.85	0.63	−0.02	−0.05	0.18^***^	0.50^***^	0.55^***^	0.61^***^	-

*N* = 348; ^*^p < 0.05, ^***^ *p* < 0.001.

Then, we ran a confirmatory factor analysis to assess the discriminant validity of four self-reported measures. We compared two measurement models: the one-factor model and the hypothesized four-factor model. In the one-factor model, all of the items were loaded onto one factor. For the hypothesized four-factor model, items were loaded onto their respective hypothetical constructs. The results demonstrated that the fit index of the model was better when items were loaded onto their respective constructs (*χ*^2^/df = 2.57, CFI = 0.91, TLI = 0.90, IFI = 0.91, RMSEA = 0.07) than when all items were loaded onto one factor (*χ^2^*/df = 5.42, CFI = 0.74, TLI = 0.71, IFI = 0.74, RMSEA = 0.11). These results suggest that the measures in the model represented distinct constructs.

Table 4 (Equation 1) demonstrates a positive relationship between active SNS use and green consuming patterns (*B* = 0.43, *p* < 0.001), H1 was supported. Both the total effect [effect = 0.43, 95% CI = (0.35, 0.51)] and the direct effect [effect = 0.27, 95% CI = (0.18, 0.35)] of active SNS use on green consumption behaviors were positive and significant.

**Table 4:** Regression results of MODEL 4 and MODEL 14 in Study 2.

Variables	Equation 1 (Green consumption behaviors)		Equation 2 (Public self-awareness)		Equation 3 (Green consumption behaviors)	
	*B*	SE	*B*	SE	*B*	SE
Control variables						
Gender	−0.08	0.06	−0.07	0.08	−0.04	0.05
Education level	−0.05	0.06	−0.001	0.08	−0.05	0.05
Income level	0.07^**^	0.02	0.03	0.03	0.04	0.02
Independent variable						
Active SNS use	0.43^***^	0.04	0.52^***^	0.06	0.22^***^	0.04
Mediator						
Public self-awareness					0.11^*^	0.05
Moderator						
Impression management motivation					0.39^***^	0.05
Public self-awareness**×** Impression management motivation					0.10^***^	0.03
*R* ^2^	0.27^***^		0.21^***^		0.48^***^	

*N* = 348; ^*^p < 0.05, ^**^ *p* < 0.01, ^***^ *p* < 0.001.

As for H2, the mediation effect of public self-awareness was statistically significant in the association between active SNS use and green consumption behaviors [effect = 0.16, 95% CI = (0.12, 0.21)]. Specifically, as shown in Table 4, there was a significant and positive relationship between active SNS use and public self-awareness (Equations 2, *B* = 0.52, *p* < 0.001), as well as between public self-awareness and green consumption behaviors (Equation 3, *B* = 0.11, *p* < 0.05). Thus, the mediation effect of public self-awareness was identified, supporting H2.

**Figure 2:**
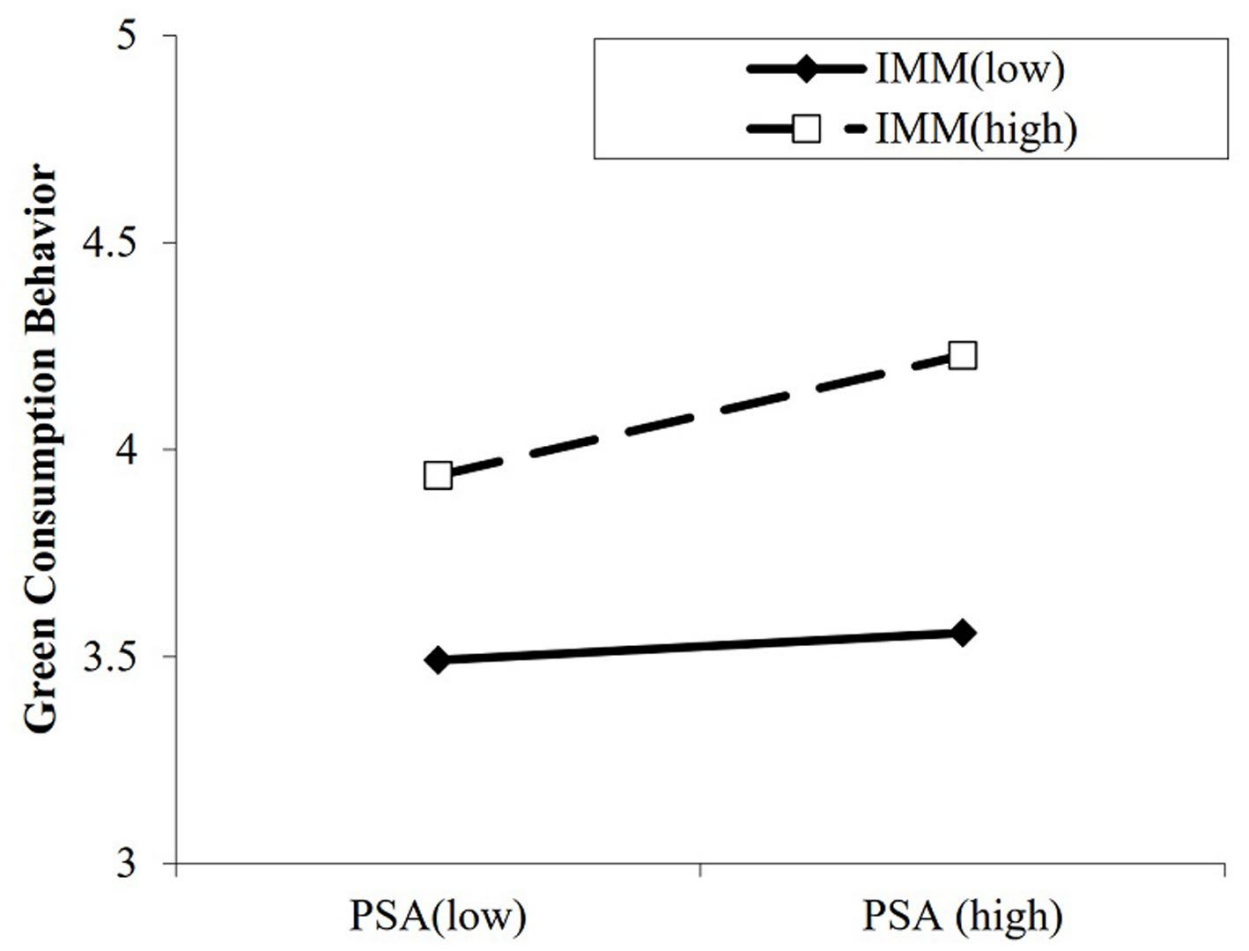
Moderating effect of impression management motivation on the relationship between public self-awareness and green consumption behaviors. PSA = public self-awareness; IMM = impression management motivation.

As for H3, Table 4 (Equation 3) demonstrates a significant positive effect of impression management motivation on green consumption behaviors (*B* = 0.39, *p* < 0.001), as well as a significant positive interaction between public self-awareness and impression management motivation on green consumption behaviors (*B* = 0.10, *p* < 0.001). The moderating role of impression management motivation on the relationship between public self-awareness and green consumption behaviors is depicted in Figure 2. Simple slopes tests demonstrated that the effect of public self-awareness on green consumption behaviors was significant for those with a high motivation for impression management [*B_simple_* = 0.18, *p* < 0.001, 95% CI = (0.07, 0.28)], but not significant for people with low impression management motivation [*B_simple_* = 0.04, *p* = 0.42, 95% CI = (−0.06, 0.14)]. Moreover, the mediation effect of public self-awareness was significant for people with high impression management motivation [effect = 0.09, 95% CI = (0.04, 0.15)], but not significant for people with low impression management motivation [effect = 0.02, 95% CI = (−0.03, 0.07)]. Taken together, the results support H3.

## Discussion

This research found a positive association between active SNS use and green consumption. Previous studies have found that SNS use can influence people’s pro-environmental behavior (Bedard and Tolmie, 2018; Pop et al., 2020; Zafar et al., 2021). Unlike previous studies, we focused on the relationship between a specific type of SNS use (active SNS use) and green consumption. By focusing on the connection between a specific type of SNS use (active SNS use) and green consumption, our study builds upon earlier researches while also expanding and deepening them. Our findings have important implications for governments, businesses, and individuals. Given that SNS has become an effective channel for promoting pro-environmental information (Chi, 2021), governments or companies can identify the target users for promotion or marketing by observing users’ SNS usage types. On the other hand, for individuals, our findings provide explanations for how individuals’ SNS use is connected with their green consumption. Besides, our study concluded that active SNS use motivates people to consume green, and those who consume green have been shown to have higher life satisfaction (Xiao and Li, 2011). Therefore, our finding enriches the literature on the positive outcomes of active SNS use by examining the impact of SNS on green consumption.

Second, our study found that public self-awareness was a mediating variable between active SNS use and green consumption. Previous studies have tested the role of SNS use on pro-environmental behavior by examining the effects of SNS use on promoting environmental responsibility (Zafar et al., 2021), altruistic and self-interested motivation (Pop et al., 2020), and perceived behavioral control (Nekmahmud et al., 2022). Unlike previous studies, our study tested public self-awareness as an explanatory mechanism for active SNS use and green consumption. Our findings are heuristically valuable to the study of self-awareness theory and provide a new perspective for understanding the relationship between SNS use and green consumption, helping to open the black box of the relationship between SNS use and pro-environmental behavior. For companies, marketers need to pay attention to the public self-awareness of social network users and try to motivate them to engage in pro-environmental purchasing behavior by using promotional tools that can stimulate their public self-awareness.

Finally, our study explored the moderating role of impression management motivation. Our study found that the effect of public self-awareness between active social network use and green consumption was significant only for individuals with high impression management motivation. As SNS has become a significant venue for self-presentation (Yang and Liu, 2017), previous studies have explored the antecedents and possible consequences of impression management on SNSs (e.g., Lee and Jang, 2019; Al-Shatti et al., 2022). This study extends the research on impression management in social networks by examining the moderating role of impression management motives in the relationship between public self-awareness motivated by active SNS use and green consumption. Governments or companies may post pro-environmental information on SNS platforms with low anonymity or strong social ties. These contexts may make users more concerned about impression management and more inclined to environmental protection (Marder et al., 2016; Lee and Jang, 2019). In addition, companies can design products with conspicuous pro-environmental symbols to encourage SNSs users to purchase.

## Limitations and future research

Our research has some limitations. First, we considered only one moderator (i.e., impression management motivation) in the relationship between active SNS use to influence green consumption through public self-awareness. Recent researches have indicated that while examining the effects of SNS use on people’s well-being or consumption behavior, the intensity of SNS use should be taken into consideration (Pellegrino et al., 2022; Valkenburg et al., 2022). Therefore, future research could consider the intensity of SNS use as a moderator of the relationship between active SNS use and public self-awareness. Such research could provide diverse suggestions for marketing practitioners to induce consumers to go green.

Second, this research found that public self-awareness mediated the relationship between active SNS use and green consumption. Recent studies suggested that social media use positively influences people’s subjective norms (Nekmahmud et al., 2022), and subjective norms positively affect sustainable behavior (Roh et al., 2022). Therefore, future research could consider the mediation role of subjective norms in the relationship between active SNS use and green consumption. This research could help better understand the correlation between active SNS use and green consumption and provide more theoretical guidance for consumers to understand their consumption behavior.

Third, the current study was conducted in China, and there may be questions about the universality of the results. Chinese collectivism encourages behavior that conforms to society. People are more likely to work together and help each other than their counterparts in individualistic western cultures, where people see themselves as independent entities, distant from their groups (Evanschitzky et al., 2014). As a person’s public self-awareness and green intention could be affected by individualism–collectivism cultural orientations (e.g., Kim and Choi, 2005; Gu and Su, 2016), the results may not generalize to countries that differ culturally from China. Future researchers might look into the connections between active SNS use and green consumption in different cultures.

## Conclusion

In the current research, we conducted two studies to examine the relationship between active SNS use and green consumption. It was found that active SNS use affects green consumption by increased public self-awareness, which is only significant for people with high impression management motivation. While most previous studies have focused on the relationship between general SNS use and pro-environmental behavior, our study takes a step forward by verifying the effect of a specific type of SNS use (active SNS use) on green consumption. Our study extends the research on the relationship between SNS use and pro-environmental behaviors and responds to the call of previous studies to refine the types of SNS use when examining its outcomes. In addition, this study extends the research on self-awareness theory and impression management theory. The results can provide guidance for governments and companies to develop more effective promotional and marketing strategies.

## Data availability statement

The raw data supporting the conclusions of this article will be made available by the authors, without undue reservation.

## Ethics statement

The studies involving human participants were reviewed and approved by Central South University Institutional Review Board. The participants provided their written informed consent to participate in this study.

## Author contributions

YG contributed to conception and design of the study, assisted with the execution of the study and data collection, and provided critical revisions. CC and YT contributed to conception and design of the study, executed the study, analyzed the data, and drafted the manuscript. DT assisted with the data collection and drafted the manuscript. All authors contributed to the manuscript revision, read, and approved the submitted version.

## Funding

The present research was supported by the Project of the National Natural Science Foundation of China (Grant No. 72072185).

## Conflict of interest

The authors declare that the research was conducted in the absence of any commercial or financial relationships that could be construed as a potential conflict of interest.

## Publisher’s note

All claims expressed in this article are solely those of the authors and do not necessarily represent those of their affiliated organizations, or those of the publisher, the editors and the reviewers. Any product that may be evaluated in this article, or claim that may be made by its manufacturer, is not guaranteed or endorsed by the publisher.
